# Linkage to HIV, TB and Non-Communicable Disease Care from a Mobile Testing Unit in Cape Town, South Africa 

**DOI:** 10.1371/journal.pone.0080017

**Published:** 2013-11-13

**Authors:** Darshini Govindasamy, Katharina Kranzer, Nienke van Schaik, Farzad Noubary, Robin Wood, Rochelle P. Walensky, Kenneth A. Freedberg, Ingrid V. Bassett, Linda-Gail Bekker

**Affiliations:** 1 Desmond Tutu HIV Centre, Institute of Infectious Disease and Molecular Medicine, Faculty of Health Sciences, University of Cape Town, Cape Town, South Africa; 2 Department of Clinical Research, Faculty of Infectious and Tropical Diseases, London School of Hygiene and Tropical Medicine, London, United Kingdom; 3 Medical Practice Evaluation Center, Department of Medicine, Massachusetts General Hospital, Boston, Massachusetts, United States of America; 4 The Institute for Clinical Research and Health Policy Studies, Tufts Medical Center, Boston, Massachusetts, United States of America; 5 Tufts Clinical and Translational Science Institute, Tufts University, Boston, Massachusetts, United States of America; 6 Department of Medicine, Faculty of Health Sciences, University of Cape Town, Cape Town, South Africa; 7 Divisions of General Medicine and Infectious Disease, Massachusetts General Hospital, Boston, Massachusetts, United States of America; 8 Division of Infectious Disease, Brigham and Women’s Hospital, Boston, Massachusetts, United States of America; 9 Harvard University Center for AIDS Research (CFAR), Boston, Massachusetts, United States of America; 10 Department of Health Policy and Management, Harvard School of Public Health, Boston, Massachusetts, United States of America; University of New South Wales, Australia

## Abstract

**Background:**

HIV counseling and testing may serve as an entry point for non-communicable disease screening.

**Objectives:**

To determine the yield of newly-diagnosed HIV, tuberculosis (TB) symptoms, diabetes and hypertension, and to assess CD4 count testing, linkage to care as well as correlates of linkage and barriers to care from a mobile testing unit.

**Methods:**

A mobile unit provided screening for HIV, TB symptoms, diabetes and hypertension in Cape Town, South Africa between March 2010 and September 2011. The yield of newly-diagnosed cases of these conditions was measured and clients were followed-up between January and November 2011 to assess linkage. Linkage to care was defined as accessing care within one, three or six months post-HIV diagnosis (dependent on CD4 count) and one month post-diagnosis for other conditions. Clinical and socio-demographic correlates of linkage to care were evaluated using Poisson regression and barriers to care were determined.

**Results:**

Of 9,806 clients screened, the yield of new diagnoses was: HIV (5.5%), TB suspects (10.1%), diabetes (0.8%) and hypertension (58.1%). Linkage to care for HIV-infected clients, TB suspects, diabetics and hypertensives was: 51.3%, 56.7%, 74.1% and 50.0%. Only disclosure of HIV-positive status to family members or partners (RR=2.6, 95% CI: 1.04-6.3, *p*=0.04) was independently associated with linkage to HIV care. The main barrier to care reported by all groups was lack of time to access a clinic.

**Conclusion:**

Screening for HIV, TB symptoms and hypertension at mobile units in South Africa has a high yield but inadequate linkage. After-hours and weekend clinics may overcome a major barrier to accessing care.

## Introduction

Sub-Saharan Africa (SSA) continues to have the highest prevalence of HIV1 and tuberculosis (TB) [2] worldwide and is also experiencing substantial non-communicable disease (NCD) epidemics of hypertension and diabetes, with the prevalence ranging from 29.2% to 48% and 1-16%, respectively [3,4,5] Given the successful roll-out of antiretroviral therapy (ART) programmes, there is now recognition that HIV services may be leveraged to provide NCD services [6-8]. Initially, HIV services were provided through vertical programmes, but due to the overlap in co-morbidities between HIV and other infectious and non-infectious diseases, integration of these services is now recommended [8].

Since the launch of South Africa’s HIV counselling and testing (HCT) campaign in April 2010, approximately 20 million individuals have been tested for HIV [9]. Given the rapid scale-up of HCT sites throughout the country and the ability of HCT programmes to reach large numbers of individuals [1], HCT may be an effective entry point for NCD screening, as screening for multiple diseases may allow a more efficient use of scarce resources [10] and increased opportunities for case-finding [8]. Furthermore, improved survival of HIV-infected patients, due to roll-out of ART combined with the increased risk in these patients for NCDs, will result in a growing burden of NCDs in this group [11]. Thus, early diagnosis of both communicable and non-communicable diseases and prompt linkage to care is critical for an effective public health response.

Studies from developing countries that integrate NCD screening programmes with HCT are scarce and were all conducted at healthcare facilities [10,12,13]. One recent study conducted in Uganda demonstrated the feasibility of integrating communicable and non-communicable disease screening in a community HIV testing campaign[14]. Mobile testing units have proven successful for the delivery of HCT [15-27] and TB screening [28] at non-healthcare facilities. Combined HIV and active TB case-finding in a mobile unit was found to be feasible [29,30] and had a high uptake and yield[30]. A recent workplace mobile screening program also showed a high yield of HIV, diabetes and hypertension diagnoses[31]. However, the success of mobile screening units depends on successful linkage to clinical care, which may be challenging.

To date, no study has evaluated a mobile unit that combines infectious and non-infectious disease screening in a resource-limited setting. The aim of this study was to assess the yield of clients newly-diagnosed with HIV, TB symptoms, diabetes and/or hypertension and linkage to HIV and other chronic disease care from a mobile testing unit. 

## Methods

### Study setting

#### Mobile testing unit

A nurse-run and counsellor-supported mobile unit provided free HCT and additional screening for TB symptoms, diabetes and hypertension to underserved urban and peri-urban townships within the Cape Metropolitan region, Western Cape, South Africa as described elsewhere [19,22,25,26,29,30]. The daily staff component comprised of one or two nurses, two or three counsellors, an educator and a driver. The unit operated five days per week at work sites (i.e. farms), outside community venues (i.e. churches, schools, halls, and sports fields), taxi-ranks and shopping centres. Sites were visited at least once every three months. Posters were placed at testing sites a day or two before the mobile unit arrival. On the day of screening staff attracted clients using flyers and a loudspeaker, furthermore brightly coloured signboards describing all the free services offered on the mobile unit were placed in and around the unit. Depending on the venue, particular groups were targeted (i.e. shopping centre and roadside: passers-by; schools: pupils and teachers). 

#### Clinical procedures

Clients were first registered by the driver using an electronic biometric fingerprint reader [26]. While clients waited to be screened, the educator conducted health talks (i.e. on sexually transmitted infections (STI), prevention of mother to child transmission, TB, diabetes, hypertension). All clients then underwent client-initiated HIV testing, according to the guidelines of the Provincial Health Government [32], followed by TB symptom and chronic disease screening. Screening was performed by a nurse and counsellor. HIV testing was conducted using the approved rapid screening test (i.e. Bioline HIV-1/2 3.0, Standard Diagnostics, Korea) which was followed by the approved rapid confirmatory test (i.e. Determine HIV-1/2, Abbott Laboratories) if the first test produced a positive result [32], according to the South African testing algorithm [33]. Newly-diagnosed HIV-infected individuals were then clinically staged, according to WHO guidelines, and had a CD4 count measured using a point-of-care (POC) machine (Alere PIMA™ Analyzer). A TB suspect was defined as a client who self-reported one or more symptoms suggestive of TB (i.e. cough or fever for >2 weeks, weight loss of >1.5 kgs in the last month, drenching night sweats, loss of appetite, hemoptysis), according to South African TB guidelines [34]. Screening for diabetes and hypertension was performed using hand-held glucometers (SD Check, Gold, Standard Diagnostics INC, South Korea) and electronic sphygmomanometers (RossMax, AW150f, Taiwan). For the purpose of this study, diabetes was defined as a random blood glucose ≥11.1 mmol/l, and hypertension was defined as a systolic blood pressure ≥160 mmHg and/or diastolic blood pressure ≥100 mmHg (“moderate hypertension”) [35]. Blood pressure was measured following questions regarding the individuals’ medical history and was taken without any prior prolonged rest in a seated position as the design of the mobile did not allow for examination couches in all rooms. If the blood pressure reading was normal (

< 140/90 mmHg), only one reading was done. If the blood pressure was ≥ 140/90mmHg, the individual was retested after several minutes of rest, on the opposite arm. Up to four blood pressure measurements were performed. The lowest blood pressure was recorded as the final result.

Individuals diagnosed with HIV, TB symptoms, diabetes and or hypertension received a referral letter written by the nurse to facilitate linkage to a healthcare facility. Referrals for other health services not offered on the mobile unit (i.e. Pap smear, STI treatment, isoniazid preventive therapy (IPT) if HIV-infected without TB symptoms, antenatal care, family planning, obesity counseling, anaemia screening or treatment, nutrition support) were also documented on this referral letter to enable clients to access care for these additional conditions. Primary healthcare clinics in this setting provide a package of care which includes both HIV, TB and other chronic disease care (i.e. diabetes and hypertension) as well as antenatal and reproductive health services [36]. For the purposes of this study, “diagnosis” of other chronic condition relates to a positive screening test (i.e. for TB symptoms, diabetes, hypertension) as a confirmed diagnosis of any one of these conditions required follow-up testing at a healthcare facility. 

Following testing, clients waited to receive one-on-one post-test counselling. HIV-negative clients received risk reduction counselling. HIV-infected clients received counselling that was informed by their POC CD4 result. This result was documented on an individual-held “Road to HIV Health” card (Figure S1) stratified by colour according to the urgency of referral to care. Clients were advised to link to care within specific intervals: clients with CD4 counts ≤200 cells/µl (ART-eligible), 201-350 cells/µl and ≥351 cells/µl were advised to link to care within one, three and six months, respectively. These time intervals were informed by the ART-eligibility criteria in the South African National guidelines at the time [37]. Clients diagnosed with TB symptoms, diabetes and/or hypertension were advised to link to care within one month of diagnosis. Contact details for all participants were documented on the mobile unit’s client locator form. On this form client’s provided details on their preferred method of contact (i.e. telephonically and/or home visits) and additional details (i.e. contact number, physical address, preferred name to use during follow-up). Clients could opt out of receiving home-visits. Only HIV-infected clients were followed-up telephonically by the counsellor one week post-diagnosis to remind the client to link to care and offer additional support as needed.

### Study design

An observational cohort study was conducted between January and November 2011. A mixed study design was used, the first component involved a retrospective review of routine clinical records to identify the yield of new diagnoses by generating a list of clients newly-diagnosed with HIV (HIV-infected cohort-primary screening service) and TB symptoms, diabetes and/or hypertension (TB and other chronic diseases cohort-additional screening services) at the mobile unit between March 2010 and September 2011. All eligible clients were sampled and then followed-up in the second component of this study to assess linkage to care after the full recommended time to link to care had passed (i.e. HIV-infected CD4 ≤200 cells/µl: 1 month post-diagnosis, HIV+ CD4: 201-350 cells/µl: 3 months post-diagnosis, HIV+ CD4: ≥351 cells/µl: 6 months post-diagnosis, and TB suspects, diabetics and hypertensives: 1 month post-diagnosis) (Table S1).

Clients eligible for follow-up had to be newly-diagnosed with any one of the above conditions, ≥18 years of age and had to provide contact details as well as consent for participation. Clients were double-counted (i.e. a client could be in the HIV-infected and TB and other chronic disease cohort). 

### Study procedures

#### Follow-up for assessment of linkage to care

Fieldworkers conducted follow-ups telephonically and/or via home visits. At least seven telephonic attempts were made for each client before a home visit. Clients who received a maximum of 10 follow-up attempts and were not traced by the end of the follow-up period were deemed “untraceable”. Client’s diagnosed with more than one condition were followed-up for each condition, after the pre-defined periods for linkage for all conditions had elapsed.

Baseline data (i.e. age, sex, previous medical history) for all mobile unit clients were available from routine client records. Additional self-reported data including socioeconomic (i.e. nationality, marital status, employment status, income, type of dwelling, level of education), health seeking behaviour (i.e. disclosure of HIV-infected status), clinical information (i.e. repeat sputum taken, chest X-ray performed, repeat glucose and/or blood-pressure reading taken, initiated ART or anti-TB/diabetes/hypertension medication) and barriers to care were obtained from the questionnaire and were only available for individuals who were traced and participated in the study. Study data were double-entered onto EpiData Entry (Version 3.1).

### Primary outcomes

We defined the following primary outcomes with linkage to care ascertained solely from client self-report:

**Yield of new diagnoses:** All clients newly-diagnosed with HIV, one or more TB symptoms, diabetes and/or hypertension between March 2010 and September 2011.
**Linked to HIV care:** A newly-diagnosed HIV-infected subject who visited the clinic and was seen by a doctor, nurse or counsellor at the healthcare facility for their referred condition as indicated on their referral letter from the mobile unit, at least once after diagnosis, within the following specific time-frames: (CD4 ≤200 cells/µl: ≤1 month post-diagnosis, 201-350 cells/µl: ≤3 months post-diagnosis, ≥351 cells/µl: ≤6 months post-diagnosis).
**Linked to TB, diabetes and/or hypertension care:** A TB suspect or subject newly- diagnosed with diabetes and/or hypertension who visited the clinic and was seen by a doctor, nurse or counsellor at the healthcare facility for their referred condition as indicated on their referral letter from the mobile unit, at least once within one month post-diagnosis.

Individuals who were deceased, untraceable and those who attempted but failed to link to care, never linked to care or linked to care but not within the pre-defined period were categorised as not having linked to care. If a client was followed-up for multiple conditions, linkage to care was assessed individually for each condition. 

### Sensitivity analysis

Additional analysis was conducted by investigating whether a client ever accessed care (i.e. any time prior to follow-up) at any healthcare facility at least once, regardless of the time-frame for linkage. 

### Statistical analysis

Analyses were conducted using STATA (Version 11.0, Stata Corporation. LP, College Station, TX, USA). Newly-diagnosed HIV-infected individuals with POC CD4 counts ≥351 cells/µl were underrepresented in the sample as only those diagnosed in 2010 were enrolled into the study and thus 157 (52%) of those diagnosed (300) were sampled. Alternatively, HIV-infected individuals with POC CD4 counts < 350 cells/µl were oversampled as those diagnosed in 2010 and 2011 were enrolled. Thus to minimise this sampling bias, sampling weights for each CD4 count stratum were calculated and the total proportions and means for each variable were adjusted for these sample weights using the survey design command. Risk ratios were assessed using Poisson regression to determine clinical and socio-demographic factors predicting linkage to care. Models for each condition (i.e. HIV-infected, TB symptoms, diabetes and hypertension) were built separately. Factors found to be associated with linkage in the univariate analysis, at a 0.1 significance level, were advanced to a multivariate analysis.

### Ethics

Informed consent was obtained from all participants. Those enrolled on the day of diagnosis in 2011 provided written consent, those diagnosed before the start of the study (2010) were followed-up telephonically and with a home visit (only if a client could not be reached telephonically and the client gave permission to be visited at home) for enrolment, and thus provided verbal and written consent, respectively. Majority of the follow-ups were conducted telephonically as this was more feasible than a home visit and most clients preferred to be contacted telephonically. The study including the consent procedure was approved by the University of Cape Town Research Ethics Committee (SA), and the Partners Healthcare Human Research Committee (Protocol 2010P002636, Boston, Massachusetts, USA).

## Results

**Figure 1:**
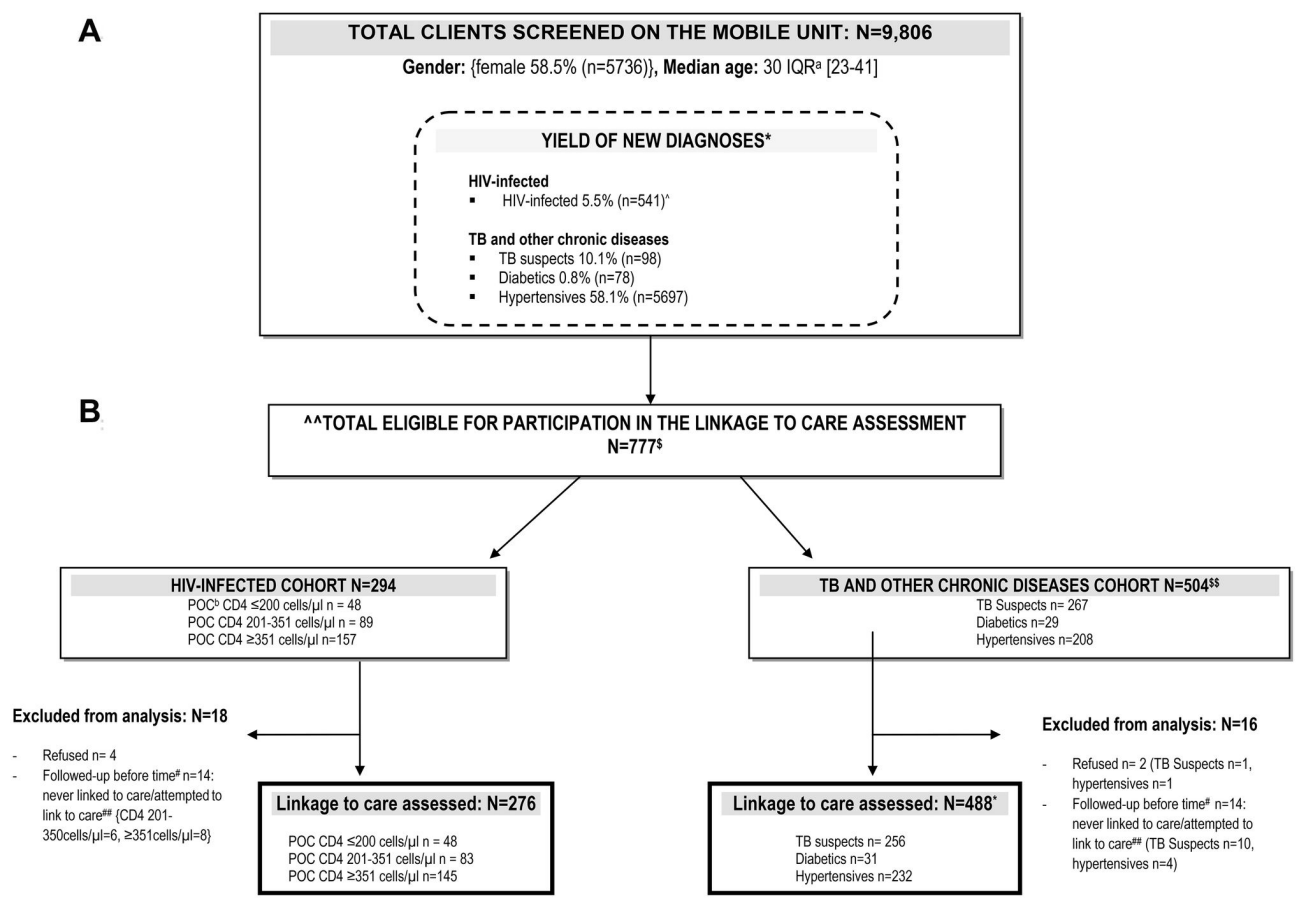
Schematic of the study inclusion and exclusion pathway. Of the 9,806 clients screened at the mobile unit between March 2010 and September 2011, 276 were eligible for follow-up in the HIV-infected cohort and 488 were eligible for follow-up in the TB and other chronic diseases cohort. 1A: ^=of the 541 newly-diagnosed HIV-infected clients, 512 (95%) had a POC CD4 count done, of whom 502 (98%) received their CD4 count result. *= The yield of new diagnoses indicates the proportion of clients screened and who were newly-diagnosed with HIV, 1 or more TB symptoms, diabetes and hypertension, in that specific hierarchical order. Individuals have not been double-counted. 1B: ^^= There was no significant difference in sex between those that were eligible and those ineligible to participate (p=0.13). $= 21 TB suspects were also newly-diagnosed with HIV. Thus the total number of clients that met the study’s eligibility criteria is less than the cumulative number of clients in the HIV-infected cohort (n=294) and TB and other chronic diseases cohort (n=504), as it excludes these 21 TB suspects from the TB and other chronic diseases cohort who are accounted for in the HIV-infected cohort. A large proportion of clients did not meet the study’s eligibility criteria due to several reasons (i.e. were HIV-negative or known HIV-positive patients, were known TB suspects/diabetics/hypertensives, were diagnosed outside the pre-specified study enrolment period, left without obtaining a referral letter or consent, had no locator details, were < 18 years);. $$= each individual is represented once in one of the three groups (i.e. TB symptoms, diabetes, hypertension); #=individuals that did not receive the full allotted time to link to care and self-reported they never linked to care or attempted but failed to link to care were excluded from the analysis.

A total of 9,806 individuals were screened at the mobile unit between March 2010 and September 2011 (Figure 1A). The majority were female (58.5%) and the median age was 30 years (interquartile range [IQR]: 23-41). The disease-specific prevalence was: newly-diagnosed HIV-infected (5.5%), TB suspects (10.1%), newly-diagnosed diabetics (0.8%) and newly-diagnosed hypertensives (58.1%). 

Of the clients newly-diagnosed, a sub-sample of 777 clients were eligible for participation in the linkage to care assessment: 294 in the HIV-infected cohort, and 504 in the TB and other chronic diseases cohort (Figure 1B). There was no significant difference in sex between those who were eligible for the linkage to care assessment and those who were not. Of the 294 clients in the HIV-infected cohort, 18 were ineligible and excluded after follow-up (refused n=4, followed-up before prescribed time n=14). A total of 276 individuals remained in the HIV-infected cohort analysis. Of the 504 clients in the TB and other chronic diseases cohort, 16 clients deemed ineligible were excluded after follow-up (refused n=2, followed-up before prescribed time n=14). A total of 488 individuals remained in this cohort, of whom 31 had more than one condition, bringing the total number for analysis to 519 (TB suspects n=256, diabetes n=31 and/or hypertension n=232). There was no significant difference in sex in each group (i.e. TB suspects, diabetics, hypertensives) compared to those who were not sampled, except for the HIV-positive group (p=0.028).

### Characteristics of clients in the HIV-infected cohort (N=276)

The majority were female (63.9%), mean age was 34.1 years and mean CD4 count was 481 cells/µl (Table 1A). Most clients (87.4%) were referred for other conditions not evaluated here. At the time of follow-up, the majority were in a relationship (73.8%) and 53.8% were unemployed (Table S2-A). Nearly two-thirds (66.3%) lived in informal dwellings and 87.8% reported having disclosed their HIV-positive status.

**Table 1:** Description of baseline clinical and socio-demographic characteristics of clients in each cohort.

**A. HIV-infected cohort (N=276)**						
**BASELINE^^^**	**Total**	**CD4 ≤200**		**CD4 201-350**		**CD4 ≥351**
	**(N=276)**	**cells/µl**		**cells/µl**		**cells/µl**
	**% (95% CI^a^)**	**(N=48) % (n)**		**(N=83) % (n)**		**(N=145) % (n)**
Age (mean years)	34.1 (32.9-35.3)	35.0		35.3		33.6
Sex (Female)*	63.9 (58.0-69.8)	60.4 (29)		48.2 (40)		68.9 (100)
WHO^b^ clinical stage (I vs^c^. II, III, IV)	57.2 (51.1-63.2)	45.8 (22)		37.8 (31)		64.6 (93)
POC^d^ CD4 count (mean cells/µl)	481 (458-505)	135		280		597
Additional morbidity						
TB^e^ suspect (vs. Known TB suspect/Known TB/Previous TB/No TB symptoms)	4.3 (2.4-6.1)	14.6 (7)		13.3 (11)		0
Newly diagnosed-diabetic (vs. Known diabetic/Not diabetic)	0	0		0		0
Newly diagnosed-hypertensive (vs. Known hypertensive/Not hypertensive)	1.9 (0.4-3.4)	4.2 (2)		4.8 (4)		0.7 (1)
Referred for other conditions (Yes)^$^	87.4 (83.2-91.7)	95.8 (46)		89.2 (74)		85.5 (124)
**B. TB and other chronic diseases cohort (N=519)**						
**BASELINE^**	**TB Suspects**		**Diabetics**		**Hypertensives**	
	**(N=256) % (n)**		**(N=31) % (n)**		**(N=232) % (n)**	
Age (median years, [IQR]^f^)	38 [31-50]		48 [38-54]		41 [34-51]	
Sex (Female)*	60.2 (154)		58.1 (18)		55.2 (128)	
Additional morbidity:						
Newly diagnosed-HIV-positive (vs. Known HIV-positive/HIV-negative)	25.4 (62)		3.2 (1)		5.7 (12)	
TB suspect (vs. Known TB suspect/Known TB patient/Previous TB/No TB symptoms)	N/A		6.3 (2)		9.1 (21)	
Newly diagnosed-diabetic (vs. Known diabetic/Not diabetic)	0.8 (2)		N/A		3.5 (8)	
Newly diagnosed-hypertensive (vs. Known hypertensive/Not hypertensive)	8.2 (21)		25.9 (8)		N/A	
Referred for other conditions (Yes)^$^	68.8 (176)		67.7 (21)		52.2 (121)	

^ baseline data from routine clinical records were available for all clients; $= conditions other than HIV, TB symptoms, diabetes, hypertension; * there was a significant difference in sex between those in the HIV cohort and those not sampled (*p*=0.028); ** there was no significant difference in sex among TB suspects (*p* value=0.49), diabetics (*p*=0.090) and hypertensives (*p*=0.752) versus those not sampled.

a= confidence interval, b=World Health Organisation, c= versus, d= point-of-care, e=tuberculosis, f=interquartile range

### Linkage to HIV care

Overall, 51.3% linked to care within the prescribed time-frame for each CD4 stratum: (≤200 cells/µl: 37.5% ≤1 month, 201-350 cells/µl: 53.0% ≤3 months, ≥351 cells/µl: 53.1% ≤6 months) (Table 2A). In the sensitivity analysis, 60.0% of participants accessed care at some point. Of the 18 ART-eligible patients that linked to care within a month, 15 (83.3%) started ART. 

**Table 2:** Study outcomes in each cohort: percentage linked to care and percentage ever linked to care.

**A. HIV-infected cohort (N=276)**						
	**Total**	**CD4 ≤200**		**CD4 201-350**		**CD4 ≥351**
	**(N=276)**	**cells/µl**		**cells/µl**		**cells/µl**
	**% (95% CI^a^)**	**(N=48) % (n)**		**(N=83) % (n)**		**(N=145) % (n)**
Linked to HIV care within pre-defined period						
Yes	51.3 (45.1-57.6)	37.5 (18)		53.0 (44)		53.1 (77)
If ART^b^-eligible, started ART		83.3 (15)		N/A		N/A
No^#^	48.7 (42.4-54.9)	62.5 (30)		47.0 (39)		47.0 (68)
Ever linked to HIV care before follow-up						
Yes	60.0 (54.2-66.5)	66.7 (32)		62.7 (52)		58.6 (85)
No^$^	40.0 (42.4-54.9)	33.3 (16)		37.3 (31)		41.4 (60)
B. TB and other chronic diseases cohort (N=519)						
	**TB^c^ Suspects**		**Diabetics**		**Hypertensives**	
	**(N=256) % (n)**		**(N=31) % (n)**		**(N=232) % (n)**	
Linked to care within pre-defined period						
Yes	56.7 (145)		74.1 (23)		50.0 (116)	
If TB suspect						
Sputum sample taken at the healthcare facility (Yes)	82.8 (120)		N/A		N/A	
Chest X-ray performed (Yes)	24.8 (36)		N/A		N/A	
Started TB treatment (Yes)	9.7 (14)		N/A		N/A	
If diabetic						
Repeat glucose test performed at the healthcare facility (Yes)	N/A		91.3 (21)		N/A	
On anti-diabetic medication (Yes)	N/A		60.9 (14)		N/A	
If hypertensive						
Repeat blood pressure test performed at the healthcare facility(Yes)	N/A		N/A		94.8 (110)	
Started anti-hypertensive medication (Yes)	N/A		N/A		65.5 (76)	
No^#^	43.3 (111)		25.9 (8)		50.0 (116)	
Ever linked to care before follow-up						
Yes	56.7 (145)		74.1 (23)		59.0 (137)	
No^$^	43.3 (111)		25.9 (8)		41.0 (95)	

# individuals that were deceased, untraceable as well as those that never linked to care, attempted but failed to link to care or linked to care but not within the time-frame; $= individuals that were deceased, untraceable, and those that never linked to care or attempted but failed to link to care.

a= confidence interval, b=antiretroviral therapy, c=tuberculosis

None of the variables examined with CD4 count (i.e. sex, age, clinical stage, referred for other conditions, nationality, employment status, marital status, monthly income, type of dwelling, highest level of schooling achieved) revealed an association with linkage to care except disclosure of HIV-positive status to family members or partners (*p*= 0.04). Controlling for CD4 count, newly-diagnosed HIV-infected individuals who had disclosed their HIV-positive status to their family member or partner were 2.6 times more likely to link to HIV care than those who had not (95% CI: 1.04-6.3).

### Characteristics of clients in the TB and other chronic diseases cohort

#### TB suspects (N=256)

The median age was 38 years and the majority were female (60.2%) (Table 1B). Over two-thirds were unemployed (68.3%) and 38.7% resided in informal dwellings (Table S2-B). Among those who linked to care (56.7%), the majority had a sputum sample taken (82.8%) (Table 2B); about one quarter had a chest X-ray done (24.8%). A total of 9.7% TB suspects who linked to care initiated TB treatment at a healthcare facility. 

#### Diabetes (N=31)

The median age was 48 years, the majority were female (58.1%) (Table 1B) and nearly two-thirds were unemployed (65.4%) (Table S2-B). Of those who linked to care (74.1%), the majority had a repeat glucose test performed (91.3%) and 60.9% commenced anti-diabetic treatment at a healthcare facility (Table 2B). 

#### Hypertension (N=232)

The median age was 41 years and more than half were female (55.2%) (Table 1B). Most clients were unemployed (54.9%) (Table S2-B). Among hypertensives who linked to care (50.0%), more than 90% had a repeat blood pressure reading and almost two-thirds (65.5%) started anti-hypertensive medication at a healthcare facility (Table 2B).

### Predictors for linkage to care for TB and other chronic diseases

No covariates for linkage to care were statistically significant in any of the three models. 

### Barriers to care

Participants reported the following as the main reasons for failing to link to HIV care: lost referral letter (18.8%), insufficient time during the day to attend a clinic (15.6%) or relocated (12.5%) (Table 3A). The majority of TB suspects, diabetics and hypertensives who failed to link to care reported having insufficient time to attend a weekday clinic due to competing priorities during the day (TB suspects 70.5%, diabetics 50.0%, hypertensives 68.9%) (Table 3B). 

**Table 3:** Barriers to care.

**A. HIV-infected cohort**						
**QUESTIONNAIRE^^**	**Total**	**CD4 ≤200**		**CD4 201-350**		**CD4 ≥351**
	**(N=211)**	**cells/µl**		**cells/µl**		**cells/µl**
	**% (95% CI^a^)**	**(N=39) % (n)**		**(N=63) % (n)**		**(N=109) % (n)**
Failed to link to care (N=42)**	15.7 (11.1-20.3)	7		11		24
Reasons for not accessing care	1 missing value					1 missing value
1. Lost referral letter	18.8 (5.3-32.2)	14.3 (1)		9.1 (1)		%1.1 (5)
2. No time to attend a healthcare facility	15.6 (4.7-26.5)	28.6 (2)		36.4 (4)		%1.1 (2)
3. Relocated to a new area	12.5 (0.8-24.2)	0		0		%1.1 (4)
**B. TB and other chronic diseases cohort**						
**QUESTIONNAIRE^^**	TB Suspects		Diabetics		Hypertensives	
	(N=208) % (n)		(N=26) % (n)		(N=204) % (n)	
Failed to link to care **	(63)		(3)		(67)	
Reasons for not accessing care	2 missing values		1 missing value		6 missing values	
1. No time to attend a healthcare facility	70.5 (43)		50.0 (1)		68.9 (42)	
2. Healthcare facility was too full and was given a date to return	6.6 (4)				6.6 (4)	
3. Lost the referral letter	3.3 (2)				4.9 (3)	

^^ data from the questionnaires were only available for clients that were traced and participated in the study;

** Indicates reported barriers to care for participants who never linked to care or attempted but failed to link to care

a= confidence interval

## Discussion

Addition of both communicable and non-communicable disease screening to existing HCT services may be effective in resource-constrained settings. This study evaluated a multiple disease screening service delivered through a mobile unit in Cape Town. Results show not only that the yield of newly-diagnosed HIV infection (5.5%) is high from this mobile unit, but also that this platform can be used to effectively identify TB suspects (10.1%) and diagnose diabetics (0.8%) and hypertensives (58.1%). While linkage to care was just over 50% for subjects in the HIV-infected cohort and TB suspects and other chronic diseases cohort, these estimates are comparable to that reported for facility-based screening [21,38-43].

Among newly-diagnosed HIV-infected clients, 51.3% linked to care within pre-defined periods. After the implementation of a POC CD4 count machine on this mobile unit, an increased proportion of clients were able to receive their CD4 results (98%) compared to when laboratory-based CD4 count testing was performed on the same unit as shown in an earlier study (73%) (*p*= 0.01) [22]. The proportion of those who ever linked to HIV care in this study (60.0%) is also higher than those ever linked to care after having received a laboratory CD4 count in a previous study (52.5%) (*p*= 0.22) (Table S3-A) [22]. A similar trend was reported on a mobile unit in Johannesburg, whereby 61% of HIV-infected clients receiving a POC CD4 result linked to care, compared to 42% who underwent laboratory CD4 count testing [44]. However, a community campaign from rural Uganda that offered clients POC CD4 testing and transport fare to a clinic reported poor linkage to HIV care (34% within three months post-diagnosis) [14]. The current study indicates the benefits of having a POC CD4 machine on a mobile unit. In addition to providing a baseline CD4 count result to nearly all clients newly-diagnosed HIV-positive on the day of diagnosis, having the client’s CD4 count result during post-test counseling assisted mobile unit nurses and counsellors in providing counseling specific to the client’s stage of infection, furthermore it allowed staff to graphically illustrate to clients on their Road to HIV Health Card (Appendix 1) where they were in their stage of infection and what HIV services should be provided to them once they link to care at their healthcare facility.

Two-thirds of ART-eligible clients ever linked to ART care (66.7%) in the current study, yet linkage to care within one month of diagnosis among this group was just over one-third (37.5%), which suggests that prompt linkage to care among ART-eligible patients remains a challenge. A recent systematic review showed that a median of 68% (range: 14%-84%) of ART-eligible patients initiate ART in SSA [45]. Our findings are consistent with the upper end of this range, as 83.3% of the 18 who linked to care started ART within one month post-diagnosis. Time to ART initiation was shorter in this study than that found in an earlier study, in which majority (69.2%) had commenced ART only within two months post-diagnosis [22]. These results indicate the combined need for intensive interventions to ensure ART-eligible patients link to care and initiate ART promptly. Such interventions could include transport vouchers, community escorts and supportive counselling as demonstrated in Tanzania [46]. Only half of the pre-ART clients linked to care within three and six month’s post-diagnosis in the CD4 201-350 cells/µl and ≥351 cells/µl strata, respectively, consistent with a study from Uganda which showed that a high baseline CD4 count (≥250 cells/µl) was among the risk factors for not enrolling into HIV care [47]. Possible interventions which could improve linkage to care among pre-ART clients include provision of medical (i.e. cotrimoxazole [48], contraception) and non-medical incentives (food hampers) [49] or psycho-social support (peer support) [50]. 

We found that disclosure of one’s newly-diagnosed HIV-positive status to a family member or partner was significantly associated with linkage to care. Disclosure was also found to be a predictor for linkage to care [51] and ART uptake [52] in studies conducted in Kenya. It is plausible that those who encounter less stigma are more likely to disclose their status than those who do not and thus are also more comfortable with accessing care. Alternatively, participants who accessed care and received intensive pre-ART or ART counselling may have been encouraged to disclose their HIV-positive status. 

This study showed that the yield of TB suspects was high and comparable to the yield of newly-diagnosed HIV-infected clients and is expected to be markedly higher if the revised national TB screening criteria is employed which includes cough of any duration [53]. Just over half of the TB suspects accessed care within one month after screening, which is consistent with a study conducted in rural Uganda which reported 52% linkage to TB care [14]. However, this is a public health concern as those who had TB and failed to link within the recommended time could have potentially transmitted TB in their communities. Noteworthy is that of the TB suspects who linked to care in this study a high proportion had sputum taken at the healthcare facility (82.8%) and10% initiated TB treatment. This is in line with current WHO guidelines, which assume that national TB programmes in resource-limited settings should screen an average of 10 suspects to identify one smear-positive case [54].

The proportion of clients newly-diagnosed with hypertension in our study (58.1%) was substantially higher than that reported in a Kenyan study (30%)[12]. In South Africa, most studies have assessed hypertension at blood pressure readings ≥140/90 mmHg and have found the prevalence to be in the range of 30-55% [55-57]. The hypertension epidemic in South Africa has been largely attributed to the increase in urbanisation and subsequent adoption of poor eating habits (i.e. high salt intake) and lack of physical activity[58-63]. The high hypertension prevalence documented in our study is likely reflective of this trend as well. Linkage to care among those newly-diagnosed with hypertension in our study (50.0%) was similar to that reported in a Ugandan study (43%) [14], and of those who linked to care; the majority had repeated blood pressure measurements and commenced anti-hypertension medication (65.5%). Linkage to care among newly-diagnosed diabetics was slightly higher in this study (74.1%) compared to that found in a study from Uganda (61%) [14], with 60.9% who had started on anti-diabetic medication. 

Not having time during the day to visit a clinic was a commonly stated barrier to care for all groups, and has been reported in other studies from this region [64]. Clinics that operate after-hours and on weekends could help to circumvent this problem. The loss of one’s referral letter and relocation were the main barriers to care in the HIV-infected cohort. These findings point to the need for counsellors to emphasise to clients the value of a referral letter for accessing care in any location and that one can access care even if the letter is misplaced. Furthermore, it highlights the need for mobile testing programmes to link their newly-diagnosed HIV-infected clients with community healthcare workers who can support clients in their entry to care [46].

This study is subject to several limitations. A larger proportion of clients screened did not meet the strict eligibility criteria for the assessment of linkage to care due to several reasons (i.e. were HIV-negative or were a known HIV-infected individual/TB suspect/diabetic/hypertensive client, were newly-diagnosed outside the pre-specified study enrolment period, left without obtaining a referral letter or consent, had no locator details), and thus linkage was only assessed in 10% (777) of those screened. Sampling bias might have been introduced as it was not feasible to sample all HIV-infected subjects in the CD4 ≥201 cells/µl stratum newly-diagnosed in 2011, since follow-up could only commence once the recommended time-frame for linkage had lapsed (i.e. after three to six months post- diagnosis). However, total proportions were calculated based on sample weighting. The small sample size in the CD4 ≤200 cells/µl stratum and the diabetes group resulted in limited power to detect associations. Clients diagnosed in 2010 were followed-up almost 10 months post-diagnosis, which made tracing challenging. More than 10% in each disease group were untraceable despite multiple attempts (HIV cohort 22%, TB suspects=18%, diabetes=16%, hypertension=12%). Misclassification of an individual as hypertensive cannot be excluded and might have resulted in a biased prevalence estimate, furthermore misclassification of linkage to care, additional follow-up tests and initiation of treatment at the healthcare facility may have also occurred as these data were ascertained through participant self-reports. Nevertheless, a previous study by our group showed that the sensitivity of self-reported linkage to care is good [22]. Recall and social desirability bias may have also influenced findings pertaining to disclosure, clinic visit date and barriers to care. Data on the telephonic follow-ups conducted by the mobile unit counsellors one week post-diagnosis was of sub-optimal quality and thus the association of successful contact on linkage to care could not be assessed. Follow-up of clients in our previous study several months after diagnosis did prove to be challenging as often locator details were missing and field staff were limited. However, following the previous study mobile staff were trained to obtain detailed locator details (i.e. alternate address, contact number, test mobile numbers on site) and thus the exact methods of follow-up were used on this study and more fieldworkers were employed.

This study also has several strengths. Trained and experienced counsellors conducted follow-ups (telephone calls and/or home visits) to ascertain outcomes in the clients preferred language to minimise respondent bias. Clients received no incentive for participation. Care at this mobile unit and care received at most of the healthcare facilities participants attended was free of charge, thus these results could be generalisable to similar settings. Mobile units are now widely implemented across SSA [15-31,44], and these results can inform policy makers considering adding screening for other infectious and non-infectious diseases. The primary outcome included a pre-specified time cut-off for linkage which may allow for easier comparisons to be made with future studies [45].

In conclusion, this study suggests that it is worthwhile to add screening for TB symptoms and hypertension to existing mobile HIV testing services, as the yield for all three conditions is substantial. This study confirmed the feasibility of POC CD4 testing on mobile HIV services and demonstrated how this information could enhance post-test counseling, specifically for ART-ineligible patients, although stronger efforts are needed to encourage linkage within one month post-diagnosis among ART-eligible patients. Linkage to care for HIV, TB and other chronic conditions, although comparable to that observed at healthcare facilities, must be improved to fully realise the public health impact of earlier screening and diagnosis. More flexible clinic operating hours, emphasis on the importance of a referral letter, and linking newly-diagnosed HIV-infected clients to patient facilitators may assist clients in overcoming barriers to care. Disclosure of one’s HIV-positive status to a family member or partner was associated with better linkage to HIV care. Integrated HIV, TB symptoms and NCD screening in mobile units holds promise for expanding the scope of HIV services and the reach of primary healthcare.

## Supporting Information

Figure S1 **Road to HIV Health Card.**
(PDF)

Table S1 **Description of enrolment and follow-up.**
(DOCX)

Table S2 **Description of clinical and socio-demographic characteristics of clients upon follow-up.**
(DOC)

Table S3 **Study outcomes in each cohort in the current and previous study: percentage ever linked to care.**
(DOC)

## References

[1] World Health Organization (2011) WHO Global HIV/AIDS Response. Progress Report 2011. Available: http://www.who.int/hiv/pub/progress_report2011/hiv_full_report_2011.pdf. Accessed: 13 March 2012.

[2] World Health Organization (2011) WHO Global Tuberculosis Control Report 2011. Available: http://whqlibdoc.who.int/publications/2011/9789241564380_eng.pdf . [Accessed on 21 February2012].

[3] Dalal S, Beunza JJ, Volmink J, Adebamowo C, Bajunirwe F et al. (2011) Non-communicable diseases in sub-Saharan Africa: what we know now. Int J Epidemiol 40: 885-901. doi:10.1093/ije/dyr050. PubMed: 21527446. 21527446

[4] Hendriks ME, Wit FW, Roos MT, Brewster LM, Akande TM et al. (2012) Hypertension in sub-Saharan Africa: cross-sectional surveys in four rural and urban communities. PLOS ONE 7: e32638. doi:10.1371/journal.pone.0032638. PubMed: 22427857. 22427857 PMC3299675

[5] Hall V, Thomsen RW, Henriksen O, Henriksen O, Lohse N (2011) Diabetes in sub-Saharan Africa 1999-2011: Epidemiology and public health implications. A systematic review. BMC Public Health 11: 564. 21756350 10.1186/1471-2458-11-564 PMC3156766

[6] Rabkin M, Nishtar S (2011) Scaling up chronic care systems: Leveraging HIV programs to support non-communicable disease services. J Acquir Immune Defic Syndr 57: 87–90. doi:10.1097/QAI.0b013e31821db92a. 21857304

[7] Rabkin M, El-Sadra WM (2011) Why reinvent the wheel? Leveraging the lessons of HIV scale-up to confront non-communicable diseases. Global. Public Health 6: 247-256. 10.1080/17441692.2011.552068 21390970

[8] UNICEF (2011) Chronic care for HIV and non-communicable diseases. How to leverage the HIV experience 2011. Available: http://www.unaids.org/en/media/unaids/contentassets/documents/unaidspublication/2011/20110526_JC2145_Chronic_care_of_HIV.pdf. [Accessed: 21 February 2012].

[9] South African Government News Agency (2012) 20 million South Africans have been tested for HIV. Available: http://www.sanews.gov.za/rss/12/12092810051001 . [Accessed: 28 Sep 2012]

[10] Janssens B, Van Damme W, Raleigh B, Gupta J, Khem S et al. (2007) Offering integrated care for HIV/AIDS, diabetes and hypertension within chronic disease clinics in Cambodia. Bull World Health Organ 85: 880-885. PubMed: 18038079. 18038079 10.2471/BLT.06.036574 PMC2636263

[11] Levitt S, Steyn K, Dave J, Bradshaw D (2011) Chronic non-communicable diseases and HIV-AIDS on a collision course: relevance for health care delivery, particularly in low-resource settings-insights from South Africa. Am J Clin Nutr 94: 1690-1696. doi:10.3945/ajcn.111.019075. PMC3226022 22089433

[12] Mwangemi F, Lamptey P (2010) Integration of HIV and CVD services in Kenya [oral presentation]. HIV and Health Systems Pre-conference, Vienna, Austria.

[13] Raposo C, Mabjaia K, de Barros RB, Muiambo C, Sebastian P et al. (2008) Counseling and testing in health: a public health approach to increase access to health promotion in Mozambique. 17^th^ International AIDS Conference Mexico City, Mexico Abstract no. WEAC0102.

[14] Chamie G, Kwarisiima D, Clark TD, Kabami J, Jain V et al. (2012) Leveraging rapid community-based HIV testing campaigns for non-communicable diseases in rural Uganda. PLOS ONE 7: e43400. doi:10.1371/journal.pone.0043400. PubMed: 22916256. 22916256 PMC3423366

[15] Morin SF, Khumalo-Sakutukwa G, Charlebois ED, Routh J, Fritz K et al. (2006) Removing barriers to knowing HIV status: same-day mobile HIV testing in Zimbabwe. J Acquir Immune Defic Syndr 41: 218-224. doi:10.1097/01.qai.0000179455.01068.ab. PubMed: 16394855. 16394855

[16] Mbopi-Kéou FX, Ongolo-Zogo P, Angwafo F, Ndumbe PM, Bélec L (2007) High impact of mobile units for mass HIV testing in Africa. AIDS 21: 1994–1996. doi:10.1097/QAD.0b013e3282f006c3. PubMed: 17721120. 17721120

[17] Matovu JKB, Makumbi FE (2007) Expanding access to voluntary HIV counselling and testing in sub-Saharan Africa: alternative approaches for improving uptake, 2001-2007. Trop Med Int Health 12: 1315-1322. doi:10.1111/j.1365-3156.2007.01923.x. PubMed: 17949401. 17949401

[18] Khumalo-Sakutukwa G, Morin SF, Fritz K, Charlebois ED, van Rooyen H et al (2008) Project Accept (HPTN 043): A community-based intervention to reduce HIV incidence in populations at risk for HIV in sub-Saharan Africa and Thailand. J Acquir Immune Defic Syndr 49: 422-431. doi:10.1097/QAI.0b013e31818a6cb5. PubMed: 18931624. 18931624 PMC2664736

[19] Van Schaik N, Kranzer K, Wood R, Bekker LG (2010) Earlier HIV diagnosis: are mobile services the answer? S Afr Med J 100: 671-674. PubMed: 21080998. 21080998 10.7196/samj.4162

[20] Grabbe KL, Menzies N, Taegtmeyer M, Emukule G, Angala P et al. (2010) Increasing access to HIV counseling and testing through mobile services in Kenya: strategies, utilization, and cost-effectiveness. J Acquir Immune Defic Syndr 54: 317-323. doi:10.1097/QAI.0b013e3181ced126. PubMed: 20453819. 20453819 PMC3225204

[21] Assefa Y, Van Damme W, Mariam DH, Kloos H et al. (2010) Toward universal access to HIV counseling and testing and antiretroviral treatment in Ethiopia: looking beyond HIV testing and ART initiation. AIDS Patient Care STDs 24: 521-525. doi:10.1089/apc.2009.0286. PubMed: 20672972. 20672972

[22] Govindasamy D, van Schaik N, Kranzer K, Wood R, Mathews C et al. (2011) Linkage to HIV care from a mobile HIV screening unit in South Africa by different CD4 count strata. J Acquir Immune Defic Syndr 58: 344-352. doi:10.1097/QAI.0b013e31822e0c4c. PubMed: 21836524. 21836524 PMC3805962

[23] Ostermann J, Reddy EA, Shorter MM, Muiruri C, Mtalo A et al. (2011) Who tests, who doesn’t, and why? Uptake of mobile HIV counseling and testing in the Kilimanjaro region of Tanzania. PLOS ONE 6: e16488. doi:10.1371/journal.pone.0016488. PubMed: 21304973. 21304973 PMC3031571

[24] Wall K, Etienne K, Nizam A, Roth DL, Telfair J et al. (2012) Influence network effectiveness in promoting couples’ HIV voluntary counseling and testing in Kigali, Rwanda. AIDS 26: 217-227. doi:10.1097/QAD.0b013e32834dc593. PubMed: 22008653. 22008653 PMC3679893

[25] Nglazi M, van Schaik N, Kranzer K, Lawn SD, Wood R et al. (2012) An incentivized HIV counseling and testing program targeting hard-to-reach unemployed men in Cape Town, South Africa. J Acquir Immune Defic Syndr 59: 281–287. doi:10.1097/QAI.0b013e31823e5bee. PubMed: 22067662. 22173039 PMC3801093

[26] Kranzer K, Govindasamy D, van Schaik N, Thebus E, Davies N et al. (2012) Incentivized recruitment of a population sample to a mobile HIV testing service increases the yield of newly diagnosed cases, including those in need of antiretroviral therapy. HIV Med 13: 132-137. doi:10.1111/j.1468-1293.2011.00947.x. PubMed: 22103326. 22103326 PMC3801091

[27] Maheswaran H, Thulare H, Stanistreet D Tanser F, Newell ML (2012) Starting a home and mobile HIV testing service in a rural area of South Africa. J Acquir Immune Defic Syndr 59: 43–46. doi:10.1097/01.qai.0000413794.33909.ca. 10.1097/QAI.0b013e3182414ed7 PMC4239475 22107821

[28] Corbett EL, Bandason T, Duong T, Eliacin L, Marchand C et al. (2010) Comparison of two active case-finding strategies for community-based diagnosis of symptomatic smear-positive tuberculosis and control of infectious tuberculosis in Harare, Zimbabwe (DETECTB): a cluster randomised trial. Lancet 376: 1244–1253. doi:10.1016/S0140-6736(10)61425-0. PubMed: 20923715. 20923715 PMC2956882

[29] Kranzer K, Olson L, van Schaik N, Raditlhalo E, Hudson E et al. (2011) Quality of induced sputum using a human-powered nebulizer in a mobile human immunodeficiency virus testing service in South Africa. Int J Tuberc Lung Dis 15: 1077–1081. doi:10.5588/ijtld.10.0684. PubMed: 21740671. 21740671

[30] Kranzer K, Lawn SD, Meyer-Rath G, Vassall A, Raditlhalo E et al. (2012) Feasibility, yield and cost of active tuberculosis case finding linked to a mobile HIV testing service in Cape Town, South Africa. PLOS Med 9:e1001281. 22879816 10.1371/journal.pmed.1001281 PMC3413719

[31] Guariguata L, de Beer I, Hough R, Bindels E, Weimers-Maasdorp D et al. (2012) Diabetes, HIV and other health determinants associated with absenteeism among formal sector workers in Namibia. BMC Public Health 12: 44. doi:10.1186/1471-2458-12-44. PubMed: 22257589. 22257589 PMC3269375

[32] Cape Western Department of Health (2009) HIV counselling and testing protocol Available: http://dws.wcape.gov.za/pls/dmsv525/PubShowFolders?p_folder_id=17541. [Accessed: 15 February 2011].

[33] The National HIV Counselling and Testing Campaign Strategy (2010) Policy and guidelines. Available: http://www.westerncape.gov.za/other/2010/6/hct_campaign_strategy_2_3_10_final.pdf. [Accessed: 15 February 2011]. .

[34] The South African Tuberculosis Control Programme (2004) Available: http://www.kznhealth.gov.za/chrp/documents/Guidelines/Guidelines%20National/Tuberculosis/SA%20TB%20Guidelines%202004.pdf. [Accessed: 17 August 2013].

[35] Joint National Hypertension Guideline Working Group (2006) African hypertension guidelines Part 2. Available: http://www.samj.org.za/index.php/samj/article/view/1111/563. [Accessed: 29 October 2011].

[36] Cape Western Government (2010) Comprehensive service plan for the implementation of healthcare Available: http://www.westerncape.gov.za/Text/2007/7/may15,2007-csp_2.pdf. [Accessed: 04 August 2013].

[37] National Department of Health South Africa (2010) Clinical guidelines for the management of HIV and AIDS in adults and adolescents. Available: http://www.doh.gov.za/docs/factsheets/guidelines/adult_art.pdf. [Accessed 29 October 2011].

[38] Mulissa Z, Jerene D, Lindtjørn B (2010) Patients present earlier and survival has improved, but pre-ART attrition is high in a six-year HIV cohort data from Ethiopia. PLOS ONE 5: e13268. doi:10.1371/journal.pone.0013268. PubMed: 20949010. 20949010 PMC2952597

[39] Micek MA, Gimbel-Sherr K, Baptista AJ, Matediana E, Montoya P et al. (2009) Loss to follow-up of adults in public HIV care systems in central Mozambique: identifying obstacles to treatment. J Acquir Immune Defic Syndr 52: 397-405. doi:10.1097/QAI.0b013e3181ab73e2. PubMed: 19550350. 19550350 PMC2784145

[40] Wanyenze RK, Hahn JA, Liechty CA, Ragland K, Ronald A et al. (2011) Linkage to HIV care and survival following inpatient HIV counseling and testing. AIDS Behav 15: 751-760. doi:10.1007/s10461-010-9704-1. PubMed: 20431933. 20431933 PMC3082586

[41] Wanyenze R, Bangsberg D, Liechty C, Nansubuga J, Kasakye H et al. (2011) Linkage to care and mortality at 18 month follow-up in a cohort of newly-diagnosed HIV-positive inpatients in Mulago Hospital, Uganda. 16^th^ International AIDS Conference Toronto, Canada Abstract no.Th PE0207

[42] MacPherson P, Corbett EL, Makombe SD, van Oosterhout JJ, Manda E et al. (2012) Determinants and consequences of failure of linkage to antiretroviral therapy at primary care level in Blantyre, Malawi: a prospective cohort study. PLOS ONE 7: e44794. doi:10.1371/journal.pone.0044794. PubMed: 22984560. 22984560 PMC3439373

[43] Kayigamba FR, Bakker MI, Fikse H, Mugisha V, Asiimwe A et al. (2012) Patient enrolment into HIV care and treatment within 90 days of HIV diagnosis in eight Rwandan health facilities: A review of facility-based registers. PLOS ONE 7: e36792. doi:10.1371/journal.pone.0036792. PubMed: 22606289. 22606289 PMC3350468

[44] Larson BA, Bistline K, Ndibongo B, Xulu T, Brennan A et al. (2012) Rapid point-of-care CD4 testing at mobile HIV testing sites to increase linkage to care: an evaluation of a pilot program in South Africa. J Acquir Immune Defic Syndr 61: e13-e17. doi:10.1097/QAI.0b013e31825eec60. PubMed: 22659650. 22659650 PMC3458178

[45] Rosen S, Fox MP (2011) Retention in HIV care between testing and treatment in sub-Saharan Africa: A systematic review. PLOS Med 8:e1001056 PubMed: 21811403. 21811403 10.1371/journal.pmed.1001056 PMC3139665

[46] Nsigaye R, Wringe A, Roura M, Kalluvya S, Urassa M et al. (2009) From HIV diagnosis to treatment: evaluation of a referral system to promote and monitor access to antiretroviral therapy in rural Tanzania. J International AIDS Society 12:1-9. PubMed: 19906291. 10.1186/1758-2652-12-31 PMC2788344 19906291

[47] Nakigozi G, Makumbi F, Reynolds S, Galiwango R, Kagaayi J et al. (2011) Non-enrolment into a free HIV care program: findings from a population-based study in Rakai, Uganda. AIDS Care 23: 764-770. doi:10.1080/09540121.2010.525614. PubMed: 21293989. 21293989 PMC3722430

[48] Kohler PK, Chung MH, McGrath CJ, Benki-Nugent SF, Thiga JW et al. (2011) Implementation of free cotrimoxazole prophylaxis improves clinic retention among antiretroviral therapy-ineligible clients in Kenya. AIDS 25: 1657-1661. doi:10.1097/QAD.0b013e32834957fd. PubMed: 21673562. 21673562 PMC3383052

[49] Kundu CK, Samanta M, Sarkar M, Bhattacharyya S, Chatterjee S et al. (2012) Food supplementation as an incentive to improve pre-antiretroviral therapy clinic adherence in HIV-positive children--experience from eastern India. J Trop Pediatr 58: 31-37. doi:10.1093/tropej/fmr026. PubMed: 21421550. 21421550

[50] Muhamadi L, Tumwesigye NM, Kadobera D, Marrone G, Wabwire-Mangen F et al. (2011) A single-blind randomized controlled trial to evaluate the effect of extended counseling on uptake of pre-antiretroviral care in Eastern Uganda. Trials 12: 184-. PubMed: 21794162. 21794162 10.1186/1745-6215-12-184 PMC3170867

[51] Amolloh M, Medley A, Owuor P, Audi B, Sewe M et al. (2011) Factors associated with early uptake of HIV care and treatment services after testing HIV-positive during home based testing and counseling (HBTC) in rural Western Kenya. 18^th^ Conference of Retroviruses and Opportunistic Infections. Boston, United States of America. Abstract no. 1077 10.1007/s10461-012-0344-5 23076720

[52] Nolan M, Gips A, Phillips L, Kweyu N, Simba M et al. (2011) Mothers2mothers - Kenyan clients who are supported to disclose their status and access support have improved PMTCT outcomes towards achieving virtual elimination of pediatric HIV/AIDS. 19^th^ International AIDS Conference Rome, Italy Abstract no. TUPE302

[53] Health Department: Republic of South Africa (2013) The South African Antiretroviral Therapy Guidelines. Available: http://www.kznhealth.gov.za/medicine/2013_ART_Guidelines.pdf. Accessed 27 May 2013.

[54] International Union Against Tuberculosis and Lung Disease (2000) Technical Guide for Sputum Examination for Tuberculosis by Direct Smear Microscopy, Fifth Edition Available: http://www.tbrieder.org/publications/books_english/microscopy.pdf. Accessed: 25 August 2013.

[55] Kandala NB, Tigbe W, Manda SO, Stranges S (2013) Geographic variation of hypertension in Sub-Saharan Africa: a case study of South Africa. Am J Hypertens 26: 382-391. PubMed: 23382489. 23382489 10.1093/ajh/hps063

[56] Thorogood M, Connor MD, Tollman SM, Hundt GL, Fowkes G et al. (2007) A cross-sectional study of vascular risk factors in a rural South African population: data from the Southern African Stroke Prevention Initiative (SASPI). BMC Public Health 7: 326. doi:10.1186/1471-2458-7-326. PubMed: 17999764. 17999764 PMC2206028

[57] Connor M, Rheeder P, Bryer A, Meredith M, Dubb A et al. (2005) The South African stroke risk in general practice study. S Afr Med J 95: 334-339. PubMed: 15931448. 15931448

[58] Steyn K, Bradshaw D, Norman R, Laubscher R (2008) Determinants and treatment of hypertension in South Africans: the first Demographic and Health Survey. S Afr Med J 98: 376-380. PubMed: 18637309. 18637309

[59] Steyn K, Fourie J, Lombard C, Katzenellenbogen J, Bourne L et al. (1996) Hypertension in the black community of the Cape Peninsula, South Africa. East Afr Med J 73: 758-763. PubMed: 8997869. 8997869

[60] Bertram Y, Steyn K, Wentzel-Viljoen E, Tollman S, Hofman J (2012) Reducing the sodium content of high-salt foods: Effect on cardiovascular disease in South Africa. S Afr Med J 109: 743-745. PubMed: 22958695. 10.7196/samj.5832 22958695

[61] Malaza A, Mossong J, Bärnighausen T, Newell ML (2012) Hypertension and obesity in adults living in a high HIV prevalence rural area in South Africa. PLOS ONE 7: e47761. doi:10.1371/journal.pone.0047761. PubMed: 23082211. 23082211 PMC3474786

[62] Peltzer K, Phaswana-Mafuya N (2013) Hypertension and associated factors in older adults in South Africa. Cardiovasc J Afr 24: 67-72. PubMed: 23736129. 23736129 10.5830/CVJA-2013-002 PMC3721893

[63] Human Sciences Research Council (2013) South African National Health and Nutrition Examination Survey. Media Release No. 1. Available: http://www.hsrc.ac.za/uploads/pageContent/3895/01%20NON-COMMUNICABLE%20DISEASES.pdf. [Accessed: 17 August 2013].

[64] Govindasamy D, Ford N, Kranzer K (2012) Risk factors, barriers and facilitators for linkage to antiretroviral therapy care: a systematic review. AIDS 26: 2059-2067. doi:10.1097/QAD.0b013e3283578b9b. PubMed: 22781227. 22781227

