# Supplementary figures and images for: Linkage to HIV, TB and Non-Communicable Disease Care from a Mobile Testing Unit in Cape Town, South Africa
Source: PLoS One. 2013 Nov 13;8(11):e80017. doi: 10.1371/journal.pone.0080017 (PMC3827432; doi:10.1371/journal.pone.0080017)

# The Road to Positive Health

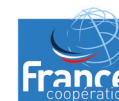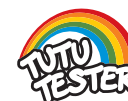

TB START/STOP  
EXAMPLE

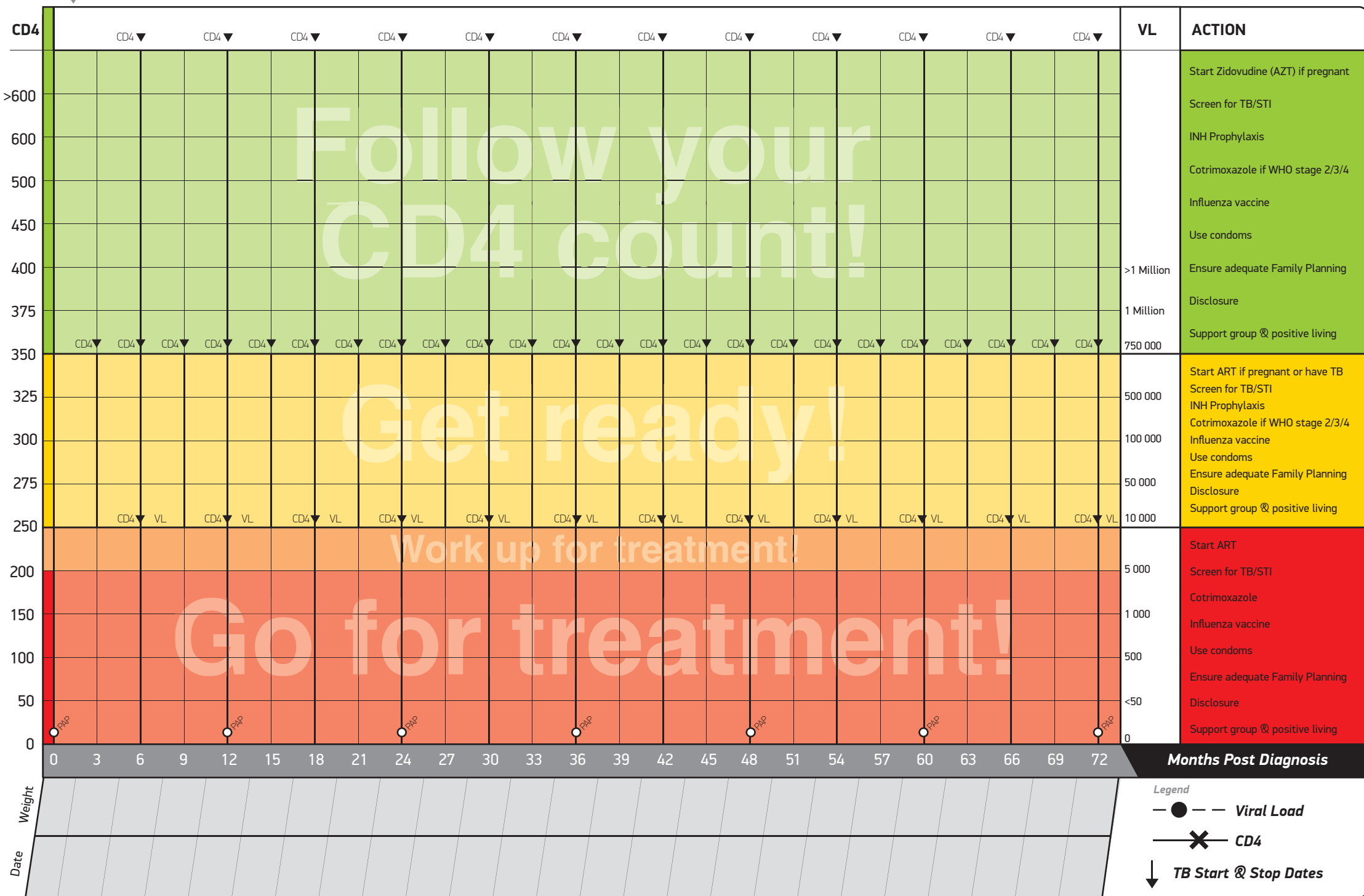

[illegible][illegible][illegible]

Supplement: Figure S1 — Road to HIV Health Card. (PDF) [file pone.0080017.s001.pdf]
