# Supplementary material for: Linkage to HIV, TB and Non-Communicable Disease Care from a Mobile Testing Unit in Cape Town, South Africa
Source: PLoS One. 2013 Nov 13;8(11):e80017. doi: 10.1371/journal.pone.0080017 (PMC3827432; doi:10.1371/journal.pone.0080017)
Supplement: Table S1 — Description of enrolment and follow-up. (DOCX) [file pone.0080017.s002.docx]

| **Condition** | **Diagnosed** | **Enrolled into the study** | **Time allocated for linkage** | **Period of follow-up to assess linkage to care** |
| --- | --- | --- | --- | --- |
| Newly-diagnosed HIV-infected  (CD4: ≤200, 201-350, ≥351 cells/µl) | 2010 | January-September 2010 | CD4: ≤200 cells/µl: 1 month post-diagnosis  CD4 201-350 cells/µl: 3 months post-diagnosis  CD4 ≥351 cells/µl: 6 months post-diagnosis | January-September 2011 |
| Newly-diagnosed HIV-infected (CD4 ≤200 cells/µl) | 2011 | January-September 2011 | 1 month post-diagnosis | February-November 2011 |
| Newly-diagnosed HIV-infected (CD4 201-350 cells/µl) | 2011 | January-July 2011 | 3 month post-diagnosis | April-November 2011 |
| Newly-diagnosed with ≥1 TB symptom/s | September 2010-September 2011 | September 2010-September 2011 | 1 month post-diagnosis | January-November 2011 |
| Newly-diagnosed diabetic | 2011 | January-September 2011 | 1 month post-diagnosis | February-November 2011 |
| Newly-diagnosed hypertensive | 2011 | January-September 2011 | 1 month post-diagnosis | February-November 2011 |

**Table S1. Description of enrolment and follow-up.**
