# Supplementary material for: Linkage to HIV, TB and Non-Communicable Disease Care from a Mobile Testing Unit in Cape Town, South Africa
Source: PLoS One. 2013 Nov 13;8(11):e80017. doi: 10.1371/journal.pone.0080017 (PMC3827432; doi:10.1371/journal.pone.0080017)
Supplement: Table S2 — Description of clinical and socio-demographic characteristics of clients upon follow-up. (DOC) [file pone.0080017.s003.doc]

**Table S2. Description of clinical and socio-demographic characteristics of clients upon follow-up.**

| **A. HIV-infected cohort (N=211)** | | | | | | |
| --- | --- | --- | --- | --- | --- | --- |
| **QUESTIONNAIRE^^** | **Total (N=211)**  **% (95% CI)^a^** | **CD4 ≤200 cells/µl (N=39) % (n)** | | **CD4 201-350 cells/µl (N=63) % (n)** | | **CD4 ≥351 cells/µl (N=109) % (n)** |
| Nationality (South African) | 91.9 (88.2-95.5) | 87.2 (34) | | 85.7 (54) | | 94.5 (103) |
| Marital status (Partner/Married) | 73.8 (67.6-80.0) | 61.5 (24) | | 70.0 (44) | | 77.1 (84) |
| Employed (No) | 53.8 (46.6-60.9) | 43.6 (17) | | 52.4 (33) | | 56.0 (61) |
| Has a monthly income (Yes) | 57.8 (50.7-64.9) | 66.7 (26) | | 52.4 (33) | | 57.8 (63) |
| Source of income (Casual wages vs^b^. Pension/Grant/Allowance) | 82.5 (75.1-89.9) | 84.6 (22) | | 87.9 (29) | | 80.7 (50) |
| Monthly income (<R1500) | 51.4 (41.9-60.8) | 53.9 (14) | | 48.6 (17) | | 51.6 (32) |
| Attended school (Yes) | 97.8 (95.6-100) | 100 (39) | | 98.4 (62) | | 97.3 (106) |
| Level of schooling completed (Primary vs. Secondary level) | 70.8 (63.5-78.0) | 75.9 (22) | | 72.9 (35) | | 69.5 (66) |
| Type of house (Informal vs. Formal) | 66.3 (59.5-73.0) | 61.5 (24) | | 63.5 (40) | | 67.9 (74) |
| Distance of nearest clinic (≤30 minutes walking distance from home) | 85.6 (80.6-90.7) | 89.7 (34) | | 84.1 (53) | | 85.3 (93) |
| Disclosed HIV-positive status (Yes) | 87.8 (82.6-93.0) | 85.7 (30) | | 90.1 (50) | | 87.2 (75) |
| Person disclosed HIV-positive status to (Family member/Partner vs. Other^#^) | 88.3 (82.6-93.9) | 93.3 (28) | | 94.0 (47) | | 85.3 (64) |
| Feels stigmatised after disclosing one’s HIV-positive status (No) | 88.5 (83.1-93.9) | 86.7 (26) | | 88.0 (44) | | 89.0 (65) |
| Know someone on ART^c^ (Yes) | 51.8 (44.4-59.0) | 55.3 (21) | | 49.2 (30) | | 51.9 (55) |
| **B. TB and other chronic diseases cohort (N=438)** | | | | | | |
| **QUESTIONNAIRE^^** | **TB^d^ Suspects (N=208)**  **% (n)** | | **Diabetics (N=26)**  **% (n)** | | **Hypertensives (N=204)**  **% (n)** | |
| Nationality (South African) | 96.2 (200) | | 100 (26) | | 90.2 (184) | |
| Marital status (Partner/Married) | 71.2 (148) | | 84.6 (22) | | 76.0 (155) | |
| Employed (No) | 68.3 (142) | | 65.4 (17) | | 54.9 (112) | |
| Has a monthly income (Yes) | 45.4 (94) | | 50.0 (13) | | 53.9 (110) | |
| Source of income (Casual wages vs. Pension/Grant/Allowance) | 68.5 (63) | | 69.2 (9) | | 81.7 (89) | |
| Monthly income (<R1500) | 62.0 (57) | | 53.9 (7) | | 38.0 (41) | |
| Attended school (Yes) | 95.2 (198) | | 92.3 (24) | | 93.6 (191) | |
| Level of schooling completed (Primary vs. Secondary level) | 79.5 (124) | | 84.2 (16) | | 63.8 (104) | |
| Reside in a house (Yes) | 99.5 (207) | | 100 (26) | | 100 (204) | |
| Type of house (Informal vs. Formal) | 38.7 (80) | | 69.2 (18) | | 65.0 (132) | |
| Distance of nearest clinic (≤30 minutes walking distance from home) | 79.8 (166) | | 80.8 (21) | | 83.3 (170) | |

^^= data from the questionnaires were only available for clients that were traced and participated in the study; #=friend/pastor/teacher/employer/work colleague/neighbour

a= confidence interval, b= versus, c= antiretroviral therapy, d=tuberculosis
