# Supplementary material for: Linkage to HIV, TB and Non-Communicable Disease Care from a Mobile Testing Unit in Cape Town, South Africa
Source: PLoS One. 2013 Nov 13;8(11):e80017. doi: 10.1371/journal.pone.0080017 (PMC3827432; doi:10.1371/journal.pone.0080017)
Supplement: Table S3 — Study outcomes in each cohort in the current and previous study: percentage ever linked to care. (DOC) [file pone.0080017.s004.doc]

**Table S3. Study outcomes in each cohort in the current and previous study: percentage ever linked to care.**

| **A. Current study: HIV-infected cohort (N=276)** | | | | |
| --- | --- | --- | --- | --- |
|  | **Total**  **(N=276)% (95% CI^a^)** | **CD4 ≤200 cells/µl**  **(N=48) % (n)** | **CD4 201-350 cells/µl**  **(N=83) % (n)** | **CD4 ≥351 cells/µl**  **(N=145) % (n)** |
| Ever linked to HIV care before follow-up |  |  |  |  |
| Yes (n=166) | 60.0 (54.2-66.5) | 66.7 (32) | 62.7 (52) | 58.6 (85) |
| No^$^ (n=110) | 40.0 (42.4-54.9) | 33.3 (16) | 37.3 (31) | 41.4 (60) |
| **B. Previous study: HIV-infected cohort (N=77)^22^** | | | | |
|  | **Total**  **(N=77) % (95% CI^a^)** | **CD4 ≤200 cells/µl**  **(N=13) % (n)** | **CD4 201-350 cells/µl**  **(N=42) % (n)** | **CD4 ≥351 cells/µl**  **(N=22) % (n)** |
| Ever linked to HIV care before follow-up |  |  |  |  |
| Yes (n=40) | 52.5 (39.4-65.6) | 100 (13) | 66.7 (28) | 36.4 (8) |
| No^£^ (n=37) | 47.5 (34.4-60.6) | 0 | 33.3 (14) | 63.6 (14) |

$= individuals that were deceased, untraceable, and those that never linked to care or attempted but failed to link to care. £=individuals that never linked to care

a= confidence interval
